# Low genetic diversity indicating the threatened status of *Rhizophora apiculata* (Rhizophoraceae) in Malaysia: declined evolution meets habitat destruction

**DOI:** 10.1038/s41598-020-76092-4

**Published:** 2020-11-05

**Authors:** Amelia Azman, Kevin-Kit-Siong Ng, Chin-Hong Ng, Chai-Ting Lee, Lee-Hong Tnah, Nurul-Farhanah Zakaria, Suhaila Mahruji, Khairuddin Perdan, Md-Zaidey Abdul-Kadir, Acga Cheng, Soon-Leong Lee

**Affiliations:** 1 Genetics Laboratory, Forest Biotechnology Division, Forest Research Institute Malaysia, 52109 Kepong, Selangor Darul Ehsan Malaysia; 2 Functional Omics and Bioprocess Development Laboratory, Institute of Biological Sciences, Faculty of Science, University of Malaya, 50603 Kuala Lumpur, Malaysia; 3 Forest Enforcement Division, Forestry Department of Peninsular Malaysia, Jalan Sultan Salahuddin, 50660 Kuala Lumpur, Malaysia; 4 Product Trade Section, Tariff Classification Division, Centre of Analysis for Industry and Custom Tariff, Department of Chemistry Malaysia, Jalan Sultan, 46661 Petaling Jaya, Selangor Darul Ehsan Malaysia

**Keywords:** Ecology, Genetics

## Abstract

Worldwide, many mangrove species are experiencing significant population declines, including *Rhizophora apiculata*, which is one of the most widespread and economically important species in tropical Asia. In Malaysia, there has been an alarming decline in *R. apiculata* populations driven primarily by anthropogenic activities. However, the lack of genetic and demographic information on this species has hampered local efforts to conserve it. To address these gaps, we generated novel genetic information for *R. apiculata*, based on 1,120 samples collected from 39 natural populations in Peninsular Malaysia. We investigated its genetic diversity and genetic structure with 19 transcriptome and three nuclear microsatellite markers. Our analyses revealed a low genetic diversity (mean *H*_e_: 0.352) with significant genetic differentiation (*F*_ST_: 0.315) among populations of *R. apiculata*. Approximately two-third of the populations showed significant excess of homozygotes, indicating persistent inbreeding which might be due to the decrease in population size or fragmentation. From the cluster analyses, the populations investigated were divided into two distinct clusters, comprising the west and east coasts of Peninsular Malaysia. The western cluster was further divided into two sub-clusters with one of the sub-clusters showing strong admixture pattern that harbours high levels of genetic diversity, thus deserving high priority for conservation.

## Introduction

Southeast Asia holds the greatest diversity of mangrove species in the world^1^. The mangroves, which form the habitat for hundreds of species at all levels of near-shore food webs, provide numerous ecosystems functions such as storm surge protection and carbon sequestration for climate change mitigation^2,3^. However, in recent decades, mangrove populations have plummeted owing mainly to various anthropogenic activities, which contribute to widespread mangrove forest degradation through land clearing, illegal logging and the over-exploitation of their highly valuable wood^1,4,5^. These were evident with around 16% mangrove species are in danger of extinction^6^.

Habitat fragmentation can affect the genetic structure of a species, primarily by increasing levels of genetic drift and inbreeding within populations, which consequently reduces the genetic diversity within the gene pool^7^. Given that populations with a lack of genetic diversity often exhibit a greater risk of extinction^8^, examining the genetic diversity patterns of the threatened mangrove populations will be the key to ensure their long-term survival. The patterns of genetic diversity in a population can be shaped by both natural (evolution) and environmental events such as human-induced climate change. Unveiling insights into the mechanisms underlying these patterns are crucial to ensure informed conservation and management of the threatened species.

*Rhizophora* is the most widely distributed species among all the mangroves species^9^. Among them, *Rhizophora apiculata* is a dominant commercial mangrove species that plays important ecological and economic role in Malaysia^10^. Locally known as Bakau Minyak, this species is preferred for its hard, strong and heavy wood, harvested mostly for wood chips, furniture, and charcoal^11,12^. While the population size of *R. apiculata* has been reduced dramatically over the past few decades^13^, little is known about the population genetics of this species. Previous work on the genus *Rhizophora* revealed low genetic diversity and high genetic differentiation, which seemed to be common traits for mangroves^14–18^. However, as most studies aimed to cover a large geographical area, sampling was not thorough, e.g. for Malaysia, only a few populations were chosen to represent the country (see^15,18^). The genetic information generated from a few populations is not sufficient to develop sound conservation plans for the species in Malaysia.

Here, we developed a new set of genic microsatellite markers (EST-SSR) in *R. apiculata* using next generation sequencing (NGS) approach, to assess its level of genetic diversity and population differentiation throughout Peninsular Malaysia. Microsatellite markers have been widely used for population analyses because of their co-dominant inheritance and high degree of polymorphism (for recent work see^19,20^). The markers developed in this study will provide a detailed genetic diversity database of *R. apiculata* which can facilitate the formulation of conservation guidelines in Peninsular Malaysia.

## Results

### Identification and development of microsatellite markers in *R. apiculata*

**Figure 1:**
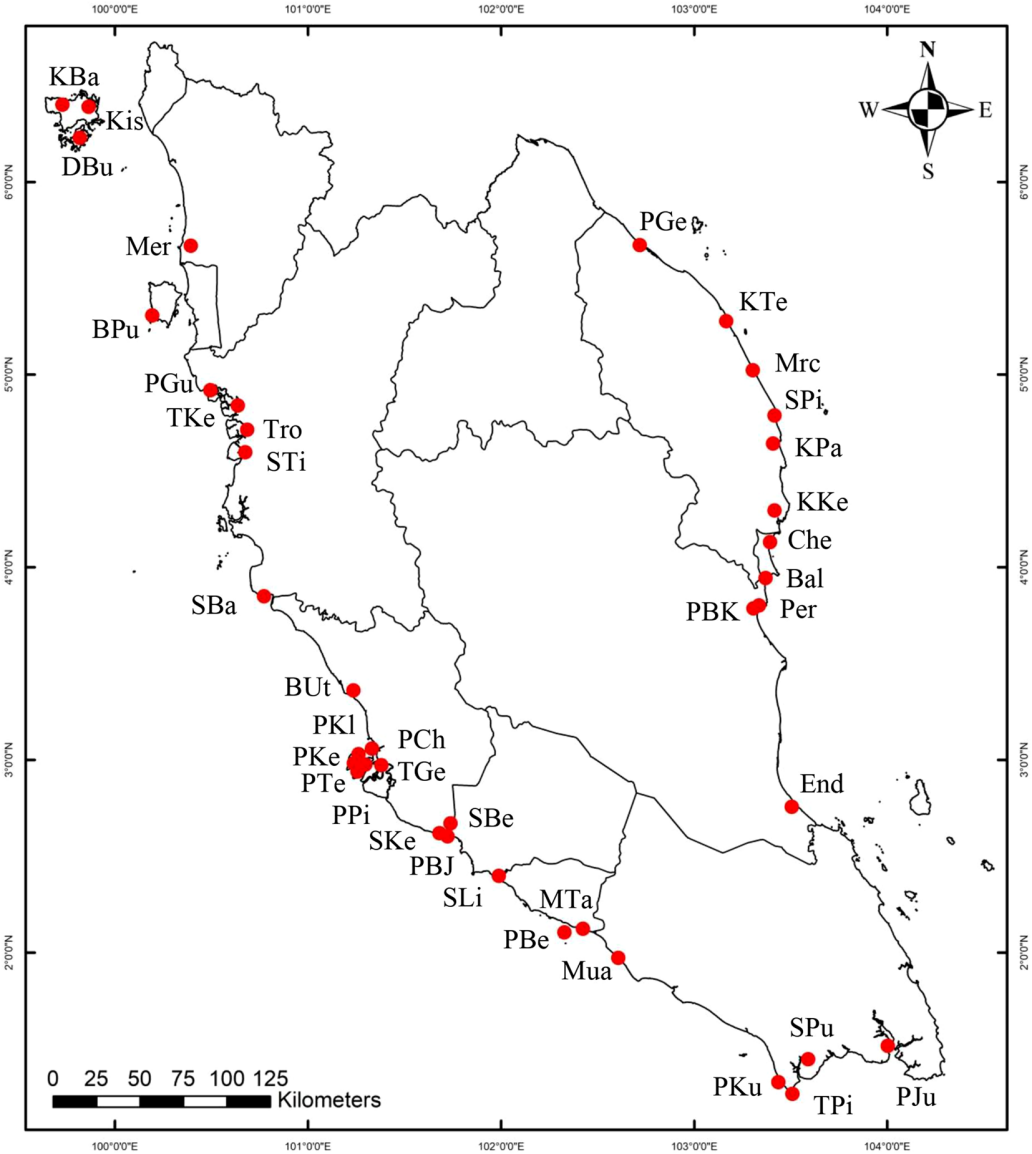
An overview of sample collection in Peninsular Malaysia. See Table 2 for detailed information on sampling sites. The map was generated using the ArcGIS v10.5 (https://desktop.arcgis.com/en/, ESRI). The Malaysian administrative boundary data for mapping was downloaded from the iGISMAP (https://map.igismap.com/share-map/export-layer/Malaysia_Polygon/cedebb6e872f539bef8c3f919874e9d7).

A total of 25,938,686 raw RNA-seq reads were generated by the Illumina HiSeq 4000 sequencer, with a total of 25,627,792 clean reads remained for further analysis after the ambiguous and low-quality sequences were filtered. These clean reads were then assembled, resulting in 141,915 contigs with an overall GC content of 44%. Using MISA software, these contigs were analysed and found to harbour 18,674 microsatellites, with the highest distribution in dinucleotide (15,898, 85.13%), followed by trinucleotide (2,403, 12.87%) and tetranucleotide microsatellites (373, 2.00%). Three highest frequencies of dinucleotide motifs that were detected were CT (16.80%), AG (16.68%) and TC (13.45%). Out of the 60 primer pairs tested, 46 yielded successful amplification. These primers were then used to screen 24 individuals of *R. apiculata* via PCR amplification and fragment analysis. Nineteen out of 46 primer pairs which showed clear genetic polymorphisms along with three other primer pairs (*RM*111, *RM*116, and *RM*121) previously developed for *R. mucronata*^21^ were selected (Table 1). The assembled contig sequences from which the newly developed microsatellite markers derived were blasted against the genome sequence of *R. apiculata*^22^ to check the reliability (Supplementary Table S1). These markers were subsequently used to genotype the 1,120 *R. apiculata* samples collected throughout Peninsular Malaysia (Fig. 1 and Table 2).

**Table 1:** Information on the 22 microsatellite markers for *R. apiculata* and the corresponding Genbank accession number.

Locus	Repeat motif	Forward primer sequence (5′–3′)	Reverse primer sequence (5′–3′)	Expected PCR product size	Genbank accession no
*Rap*T01	(AT)_10_	CCACACATTTTGCACCGAGC	CTCAATCGCCTCGGAATGA	298	MT364422
*Rap*T02	(CT)_11_	TAAAGTGCGTGAATCCGCCA	ACTGCAACTCCACCGCTTAA	138	MT364423
*Rap*T06	(CT)_14_	TCCTCCTTGCTCCAAGCTTG	ACATGGAGCCTGTAGCCAAA	349	MT364424
*Rap*T08	(GA)_16_	TGATTCACTGCAAATGAGGCC	ACGAAACAAGCTTGCTCCCT	102	MT364425
*Rap*T09	(TC)_16_	AGGCTCGGGATTTTCCTTCG	CTGGGAAGCATGGGGTAGTG	170	MT364426
*Rap*T16	(AG)_11_	GGTGGATTGAGGGAAGTGGG	GGACACCTGGCAAACCCTTA	296	MT364427
*Rap*T17	(GA)_18_	GTGGGAGCCTGCAAAGTTTG	GCGCGTTGAGCTCAGTTTAG	281	MT364428
*Rap*T18	(AT)_11_	CACAGCAGTGTCATGGGCTA	AGGAGGAAGCTCATGCACAA	165	MT364429
*Rap*T20	(CT)_12_	TCAACCGCCTTTTCCCTCTC	ACCTTAAGCAATCTCCGGCA	102	MT364430
*Rap*T21	(GA)_15_	TATGTGGTCTGTGCAACCCC	GAAGCAGGCTGGATGGACTT	156	MT364431
*Rap*T23	(AGA)_11_	TGGAGTGAAACACCTGATCCA	TGGGGCCAACAAAATGAGGA	148	MT364432
*Rap*T25	(ATT)_11_	ATGCGACGTCACTCTGGTTT	CTCCTTCAGCTCTCCGCAAA	267	MT364433
*Rap*T31	(CAT)_13_	TCGCTTAAGTCCCTGCCTTG	CCTTTGTAGGAGTGGCTGCA	217	MT364434
*Rap*T38	(AAT)_10_	ACAGCCTTTACTCCAGCGAG	AGGTAAGGAACGCCGTGTTG	347	MT364435
*Rap*T43	(CAG)_11_	GCATAGACAGGGCAGGGATC	CACCACGAGACCAAGTCTGA	153	MT364436
*Rap*T46	(CAG)_12_	CCCAATCCGAACCCTAACCC	TGCTGCACATTCTGCCCTAA	116	MT364437
*Rap*T51	(AGAC)_6_	TCTGAACCCGTTTTCTCCGG	GCCAGCAGCAAAATGAGGAC	359	MT364438
*Rap*T53	(TCTT)_6_	CCGCAACGGTTCTTCAAAGG	GACCACAACAGTAGACAGCC	338	MT364439
*Rap*T60	(AAAG)_9_	TCCACTTCGGTCTTTTCCCT	GGGCTAGAAGCGTCCGATTT	369	MT364440
*RM*111*	(TC)_13_	AACCGTTACTCGCGTATGCT	CATTGCCTCCATTCCATT	149	AB721976
*RM*116*	(TA)_12_	ATAAGACCATATGTAACACCCATTT	CCTCCTCTCATTCTTCATTTCA	152	AB721980
*RM*121*	(ATC)_12_	TGGCCTATAGAGAAAGCGGA	CCTTCAATCCCAAACAGC	179	AB721982

*Nuclear microsatellite markers developed for *R. mucronata*^21^*.*

**Table 2:** Sampling location, habitat condition and genetic diversity measures (number of alleles per locus, *A*_a_; observed heterozygosity, *H*_o_; expected heterozygosity, *H*_e_; and allelic richness, *R*_s_) and inbreeding coefficient (*F*_IS_) of the 39 populations of *R. apiculata* from Peninsular Malaysia.

No	Population (Code)	Habitat condition^a^	State	*N*	Latitude	Longitude	Genetic diversity measures				
							*A*_a_	*H*_o_	*H*_e_	*R*_s_	*F*_IS_
1	Kubang Badak (KBa)	Developed	Kedah	30	06°24'	99°43'	2.7	0.219	0.261	2.30	0.161*
2	Kisap (Kis)	Undisturbed	Kedah	30	06°23'	99°51'	4.3	0.321	0.404	3.19	0.207*
3	Dayang Bunting (DBu)	Undisturbed	Kedah	29	06°13'	99°49'	4.0	0.242	0.436	3.17	0.450*
4	Merbok (Mer)	Developed	Kedah	28	05°40'	100°23'	2.7	0.278	0.290	2.32	0.044*
5	Balik Pulau (BPu)	Developed	Pulau Pinang	28	05°18'	100°11'	2.6	0.234	0.247	2.13	0.054*
6	Pulau Gula (PGu)	Undisturbed	Perak	30	04°55'	100°29'	2.9	0.312	0.322	2.34	0.029
7	Teluk Kertang (TKe)	Logged	Perak	30	04°50'	100°38'	3.0	0.305	0.327	2.39	0.071
8	Trong (Tro)	Developed	Perak	30	04°42'	100°41'	2.8	0.250	0.308	2.31	0.191*
9	Sungai Tinggi (STi)	Undisturbed	Perak	30	04°35'	100°40'	2.9	0.274	0.301	2.24	0.091
10	Sungai Batang (SBa)	Developed	Perak	28	03°50'	100°46'	3.1	0.194	0.302	2.43	0.363*
11	Banjar Utara (BUt)	Developed	Selangor	29	03°21'	101°14'	3.0	0.227	0.281	2.35	0.193*
12	Pulau Klang (PKl)	Logged	Selangor	26	03°03'	101°19'	3.4	0.366	0.375	2.86	0.025
13	Pulau Ketam (PKe)	Developed	Selangor	26	03°01'	101°15'	4.6	0.358	0.478	3.63	0.256*
14	Pulau Tengah (PTe)	Undisturbed	Selangor	29	02°58'	101°14'	3.7	0.409	0.395	2.87	-0.035
15	Pulau Pintu Gedong (PPi)	Undisturbed	Selangor	29	02°56'	101°15'	3.7	0.353	0.355	2.83	0.007
16	Pulau Che Mat Zin (PCh)	Undisturbed	Selangor	28	02°58'	101°17'	3.6	0.377	0.386	2.89	0.022
17	Telok Gedong (TGe)	Logged	Selangor	31	02°58'	101°22'	3.6	0.322	0.364	2.76	0.118*
18	Sepang Besar (SBe)	Developed	Selangor	26	02°40'	101°44'	3.2	0.369	0.392	2.77	0.061*
19	Sepang Kecil (SKe)	Developed	Selangor	28	02°37'	101°40'	3.1	0.339	0.376	2.68	0.101*
20	Jimah (PBJ)	Undisturbed	Negeri Sembilan	30	02°36'	101°43'	3.5	0.405	0.411	2.94	0.017
21	Sungai Linggi (SLi)	Undisturbed	Negeri Sembilan	30	02°23'	101°59'	3.7	0.456	0.463	3.07	0.019
22	Pulau Besar (PBe)	Developed	Melaka	9	02°06'	102°19'	2.9	0.404	0.455	2.91	0.118*
23	Merlimau Tambahan (MTa)	Developed	Melaka	30	02°07'	102°25'	3.9	0.483	0.473	3.20	-0.023
24	Muar (Mua)	Developed	Johor	30	01°58'	102°36'	4.5	0.370	0.503	3.44	0.268*
25	Pulau Kukup (PKu)	Developed	Johor	29	01°19'	103°26'	4.6	0.373	0.498	3.61	0.256*
26	Tanjung Piai (TPi)	Developed	Johor	29	01°16'	103°30'	3.7	0.423	0.467	3.11	0.096*
27	Sungai Pulai (SPu)	Undisturbed	Johor	30	01°26'	103°35'	3.7	0.404	0.474	3.07	0.150*
28	Pulau Juling (PJu)	Undisturbed	Johor	30	01°30'	104°00'	2.9	0.328	0.366	2.49	0.106*
29	Endau (End)	Undisturbed	Pahang	30	02°45'	103°30'	2.7	0.312	0.370	2.36	0.159*
30	Kuantan (PBK)	Developed	Pahang	30	03°47'	103°18'	2.5	0.273	0.323	2.16	0.157*
31	Peramu (Per)	Developed	Pahang	30	03°48'	103°20'	2.8	0.310	0.369	2.33	0.165*
32	Balok (Bal)	Undisturbed	Pahang	30	03°56'	103°22'	2.5	0.274	0.312	2.16	0.123
33	Cherating (Che)	Developed	Pahang	30	04°07'	103°23'	2.8	0.302	0.329	2.25	0.085*
34	Kuala Kemaman (KKe)	Developed	Terengganu	30	04°17'	103°24'	2.4	0.226	0.284	2.07	0.205*
35	Kuala Paka (KPa)	Undisturbed	Terengganu	30	04°38'	103°24'	2.3	0.292	0.319	2.12	0.085*
36	Sungai Pimpin (SPi)	Undisturbed	Terengganu	30	04°47'	103°24'	2.6	0.303	0.312	2.21	0.031
37	Merchang (Mrc)	Logged	Terengganu	28	05°01'	103°18'	3.6	0.256	0.399	2.87	0.363*
38	Kuala Terengganu (KTe)	Developed	Terengganu	30	05°16'	103°09'	2.6	0.309	0.343	2.31	0.101*
39	Pengkalan Gelap (PGe)	Undisturbed	Terengganu	30	05°40'	102°43'	2.7	0.314	0.327	2.32	0.042
				1,120			3.2	0.299	0.352		

^a^*Logged* logged over for firewood, charcoal production and construction, *Developed* development for human settlement, aquaculture, ecotourism, road construction and land use for plantation, and *Undisturbed* undisturbed population.

*Significant at 95% confidence interval.

### Genetic diversity

Descriptive statistics for the 39 populations of *R. apiculata* based on the 22 polymorphic microsatellite markers are listed in Table 2. These microsatellite markers had number of alleles per locus (*A*_a_) ranging from 2.3 to 4.6 alleles for all samples, with an average of 3.2 alleles per locus. The mean values for observed heterozygosity (*H*_o_) and expected heterozygosity (*H*_e_) were 0.299 and 0.325, respectively. The expected heterozygosity (*H*_e_) at the population level ranged from 0.247 (Balik Pulau) to 0.503 (Muar). The allelic richness (*R*_s_) ranged from 2.07 to 3.63, showing marginal differences among the populations investigated. All loci deviated from Hardy–Weinberg equilibrium (HWE), with heterozygote frequencies being either lower or higher than expected (Table 2). Approximately 95.9% of the 39 tested populations had excess of homozygotes with positive inbreeding coefficient values (*F*_IS_) ranging from 0.007 to 0.450. Of that, 66.7% (26 populations) were found to be statistically significant (*p* < 0.05), mainly of the populations with disturbed habitat, either due to logging for firewood, charcoal production and construction (Telok Gedong and Merchang) or development for human settlement, aquaculture, ecotourism, road construction and land use for plantation (Kubang Badak, Merbok, Balik Pulau, Trong, Sungai Batang, Banjar Utara, Pulau Ketam, Sepang Besar, Sepang Kecil, Pulau Besar, Muar, Pulau Kukup, Tanjung Piai, Kuantan, Peramu, Cherating, Kuala Kemaman and Kuala Terengganu). There were only two populations with negative *F*_IS_ values (Pulau Tengah, *F*_IS_ = − 0.035 and Merlimau Tambahan; *F*_IS_ = − 0.023) or excess of heterozygotes but were not statistically significant (*p* < 0.05) (Table 2).

Most of the total genetic diversity (*H*_T_ = 0.532) was partitioned within population (*H*_S_ = 0.370) (Table 3). The values recorded for Wright index (*F*_ST_) ranged from 0.079 to 0.638, while those for the Slatkin’s divergence parameter (*R*_ST_), varied from 0.038 to 0.617. The proportion of genetic variation distributed among populations (*G*_ST_) was estimated at 0.305, indicating that 30.5% of genetic variability was distributed among populations (Table 3). The mean *F*_ST_ (0.315) estimate was slightly higher than *G*_ST_ and was significantly greater than zero (*p* < 0.05), while the mean *R*_ST_ (0.242) was lower than *F*_ST_ (Table 3).

**Table 3:** Genetic diversity assessment in *R. apiculata*.

Locus	*H*_T_	*H*_S_	*G*_ST_	*F*_ST_	*R*_ST_	Genbank accession no
*Rap*T01	0.489	0.403	0.176	0.180	0.249	MT364422
*Rap*T02	0.532	0.283	0.469	0.487	0.617	MT364423
*Rap*T06	0.648	0.430	0.337	0.350	0.305	MT364424
*Rap*T08	0.615	0.564	0.084	0.089	0.083	MT364425
*Rap*T09	0.818	0.645	0.212	0.221	0.400	MT364426
*Rap*T16	0.512	0.198	0.614	0.638	0.402	MT364427
*Rap*T17	0.842	0.683	0.189	0.195	0.145	MT364428
*Rap*T18	0.633	0.395	0.377	0.392	0.042	MT364429
*Rap*T20	0.518	0.456	0.121	0.126	0.070	MT364430
*Rap*T21	0.656	0.396	0.396	0.412	0.528	MT364431
*Rap*T23	0.439	0.166	0.622	0.616	0.090	MT364432
*Rap*T25	0.529	0.217	0.590	0.609	0.581	MT364433
*Rap*T31	0.560	0.432	0.230	0.229	0.270	MT364434
*Rap*T38	0.584	0.460	0.212	0.222	0.038	MT364435
*Rap*T43	0.257	0.237	0.079	0.079	0.071	MT364436
*Rap*T46	0.404	0.183	0.548	0.564	0.070	MT364437
*Rap*T51	0.470	0.275	0.415	0.431	0.273	MT364438
*Rap*T53	0.357	0.188	0.474	0.480	0.508	MT364439
*Rap*T60	0.201	0.180	0.103	0.106	0.117	MT364440
*RM*111*	0.694	0.603	0.132	0.134	0.275	AB721976
*RM*116*	0.690	0.526	0.237	0.249	0.094	AB721980
*RM*121*	0.259	0.222	0.140	0.147	0.070	AB721982
	0.532	0.370	0.305	0.315**	0.242	

*Nuclear microsatellite markers developed for *R. mucronata*^21^.

**Significant at 95% confidence interval.

### Population genetic structure

**Figure 2:**
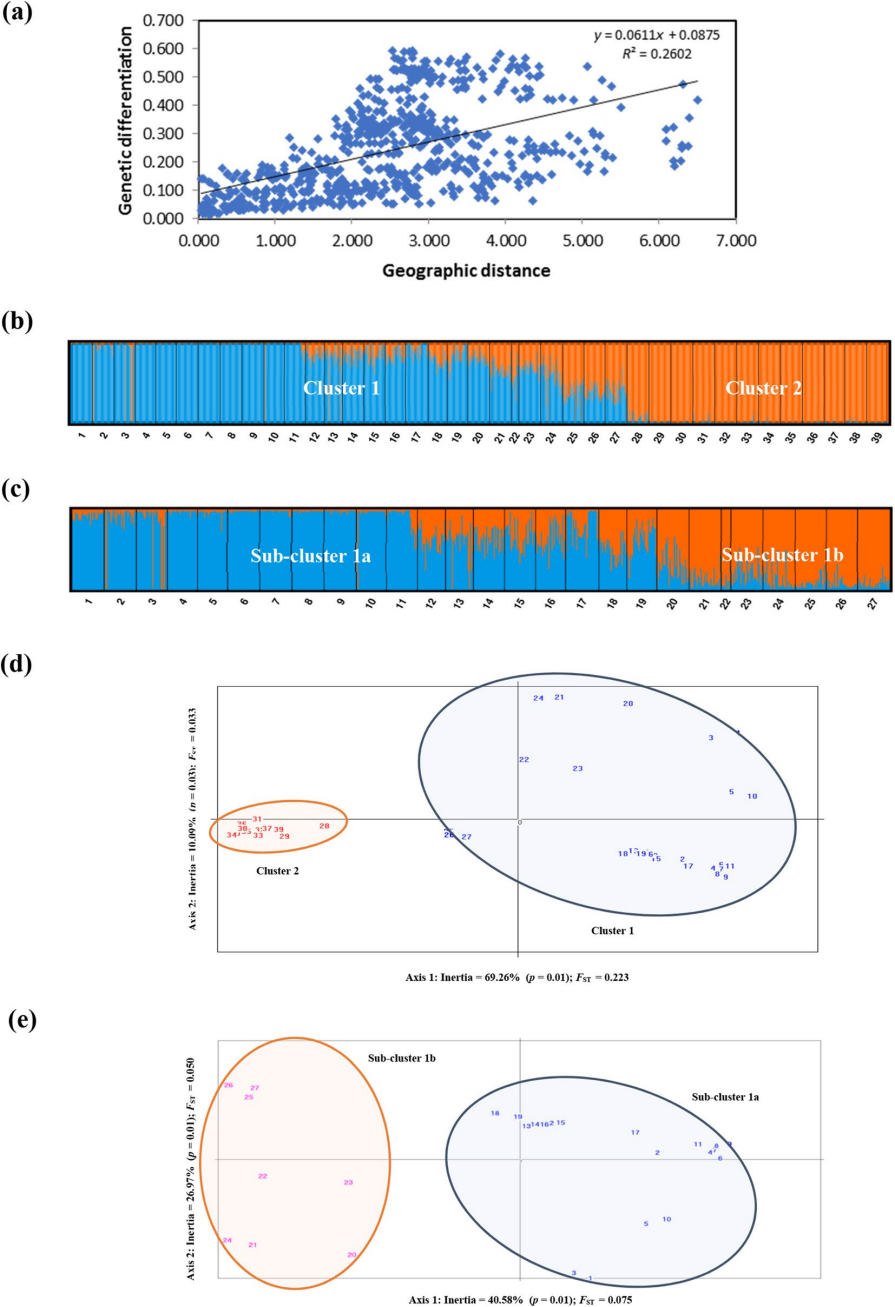
(**a**) Result of Mantel test for Isolation-by-Distance using Nei’s genetic distance; (**b**) STRUCTURE showing the division of *R. apiculata* populations into two main clusters; (**c**) Sub-structuring of Cluster 1, forming Sub-clusters 1a and 1b; (**d**) Principal component analysis (PCA) based on pairwise *F*_ST_ of 39 *R. apiculata* populations, assigning the populations into two distinct clusters that coincide with western (orange color: Straits of Malacca) and eastern (blue color: South China Sea) Peninsular Malaysia and (**e**) PCA showing Cluster 1 further divided into two sub-clusters.

Genetic differentiation between populations increased as distance between populations increased (*R*^2^ = 0.2602) (Fig. 2a). Additionally, the results from the model-based Bayesian clustering analysis using STRUCTURE v2.3.1 indicated the presence of two main clusters within Peninsular Malaysia, one formed by the populations in western Peninsular Malaysia (Cluster 1: populations 1–27), and the other by those in eastern Peninsular Malaysia (Cluster 2: populations 28–39). The STRUCTURE algorithm showed the best clustering at *K* = 2, providing good biological explanation since the clusters coincided with two geographical groups^23^ corresponding to the Straits of Malacca (western Peninsular Malaysia) and South China Sea (eastern Peninsular Malaysia) (Fig. 2b). A gradually increasing level of admixture was observed in populations between Kedah and west part of Johor (Cluster 1: populations 1–27). When genetic variation is hierarchically organised, the algorithm underlying STRUCTURE detects only the uppermost level of population structure^23^. Hence, STRUCTURE analysis within cluster level further divided western cluster into two sub-clusters: Sub-cluster 1a (populations 1–19) and Sub-cluster 1b (populations 20–27) (Fig. 2c). Interestingly, the observed strong admixture pattern in Sub-cluster 1b suggests that the populations within this sub-cluster harbour higher levels of genetic diversity and could be an important genetic reservoir for *R. apiculata* in Peninsular Malaysia.

**Figure 3:**
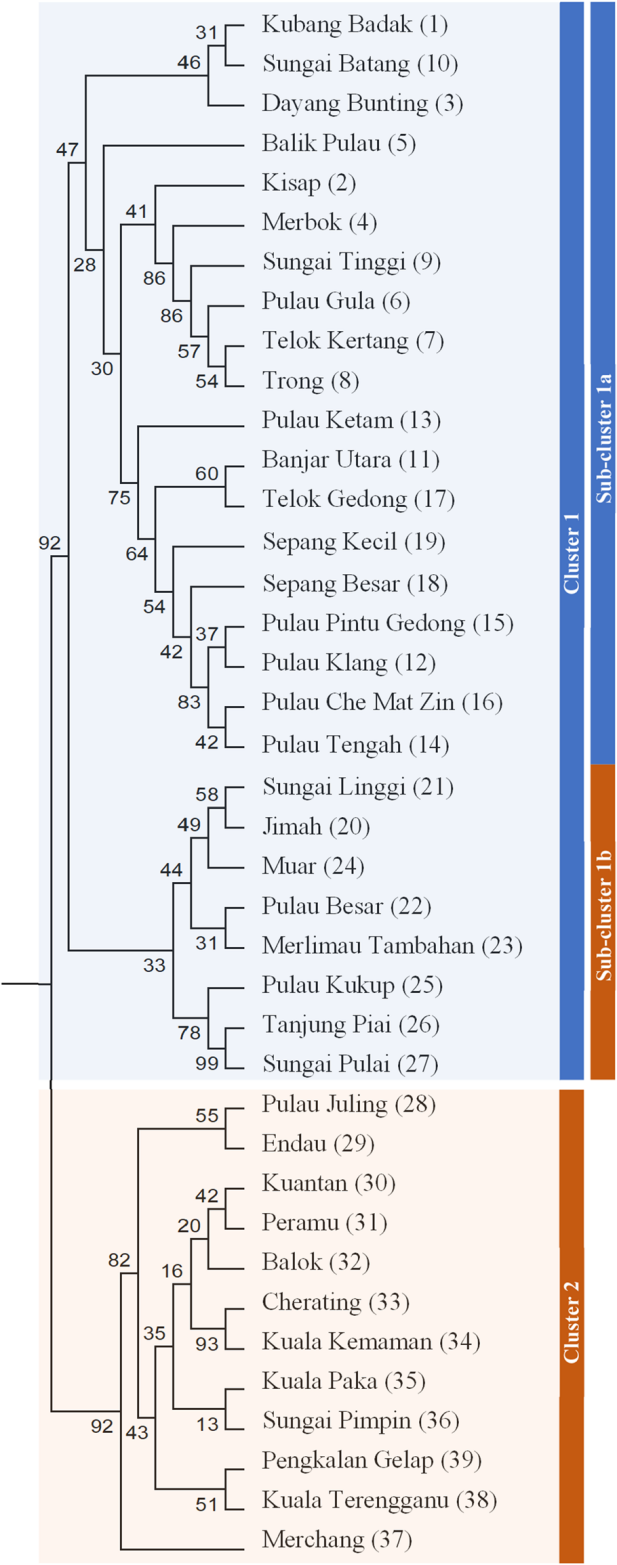
UPGMA dendrogram based on mean character differences estimated from microsatellite data of 1,120 *R. apiculata* individuals in 39 populations.

Results from the principal component analysis (PCA) corroborates with the STRUCTURE analysis, whereby the 39 populations were also divided into two distinct clusters (Fig. 2d), i.e. Cluster 1 along the Straits of Malacca (western Peninsular Malaysia) and Cluster 2 facing the South China Sea (eastern Peninsular Malaysia). Similarly, Cluster 1 was further divided into two sub-clusters (Fig. 2e). To complement the population structure analysis, a UPGMA dendrogram was constructed with MEGA v5.0 based on Nei’s *D*_A_^24^, producing two main branches that illustrate the two major clusters and two sub-clusters within Cluster 1 (Fig. 3).

When the 39 populations were grouped based on the two main clusters, AMOVA revealed that 45% of the variation was apportioned between the western and eastern regions of Peninsular Malaysia, 13% among populations within regions, and 42% within populations (Table 4).

**Table 4:** Analysis of molecular variance (AMOVA) performed by dividing the 39 populations into geographical regions.

Source of variation	Degree of freedom	Sum of squares	Variance component	Percentage of variance
Among regions	1	4950.041	9.966	45
Among populations within regions	37	3443.394	2.918	13
Within populations	1081	10,125.069	9.366	42
Total	1119	18,518.504	22.250	100

## Discussion

From our transcriptome data, we identified a total of 18,674 microsatellites, with dinucleotide repeat motifs (85.13%) being the most frequent type of microsatellite. Similar observations, whereby dinucleotides was the most abundant motif in the plants’ genomes, have been reported for other tree species such as grey mangrove (*Avicennia marina*)^25^, downy oak (*Quercus pubescens*)^26^ and crape myrtle (*Lagerstroemia* spp.)^27^. A total of 19 transcriptome-based microsatellite markers (EST-SSR) were developed in this study. To increase the number of markers for genotyping, three additional nuclear microsatellite markers (gSSR) which were previously developed for *R. mucronata*^21^ have been validated and utilised in our study. Increasing the number of polymorphic loci can greatly enhance the precision of estimates of genetic distance^28^. The high cross-species transferability of microsatellite markers between *R. apiculata* and *R. mucronata* has previously been demonstrated^9^.

Generally, low genetic diversity is common in mangrove plant species particularly the *Rhizophora* species^9,16,22^. Low levels of genetic diversity (mean *H*_e_ = 0.352, Table 2) in *R. apiculata* were observed in the present study. The result is comparable to other mangrove species such as *R. mucronata* (*H*_e_ = 0.354)^9^, *R. stylosa* (*H*_e_ = 0.321)^9^, and *Sonneratia alba* (*H*_e_ = 0.280)^29^. The low level of genetic diversity occurring within *R. apiculata* populations suggests that this species may have experienced severe habitat fragmentation or population size reduction.

Our results also showed significant excess of homozygotes or positive inbreeding coefficient values (*F*_IS_) in most of the *R. apiculata* populations, consistent with the observed habitat destruction found in most of these populations (Table 2). Hence, the observed positive *F*_IS_ values might be the result of selfing and/or mating between close relatives due to the reduction in population size caused by the anthropogenic activities. This is also a common result when drift proceeds in small population. As drift proceeds and each population becomes different from one other, the genetic variation among populations increases^30,31^.

*R. apiculata* is considered a threatened mangrove species in Southeast Asia, and its population decline is due mainly to deforestation and land conversion^4,32,33^. The high wood density is an attractive selling factor for *R. apiculata* (0.600 gcm^-3^), causing extensive over-exploitation of the species^12,34^. Owing to this, decline in effective population size and aggravate loss of genetic diversity ultimately reduced resilience of populations to anthropogenic climate change^35^. The genetic fragility in *R. apiculata* is of concern, considering that future impacts of environmental changes, whether natural or otherwise, will likely to further reduce its genetic diversity and threaten its long-term viability.

Wright’s *F*-statistics^36^ is among the most common methods used to estimate the level of heterozygosity in a population. The *F*_ST_ is more sensitive in detecting intraspecific differentiation as compared to its analogue, the *R*_ST_^37,38^. The *R*_ST_, however, is thought to be a better predictor for interspecific divergence because it can effectively reflect the mutation patterns of microsatellites^39^. Therefore, both models were applied in the present study, revealing a strong population genetic structure of *R. apiculata* (*F*_ST_ = 0.315, *R*_ST_ = 0.242). We found that approximately 68.5% and 31.5% of genetic variations were distributed within and among *R. apiculata* populations, respectively. Other mangrove studies also revealed high population differentiations, namely in *Avicennia marina* (0.410)^25^, *Ceriops tagal* (0.529)^40^, and *A. germinans* (0.410)^41^.

All the three cluster analyses performed in this study showed similar results, whereby the 39 populations were divided into two major geographical clusters (Cluster 1: populations 1–27; and Cluster 2: populations 28–39), with strong admixture pattern observed between southern part of western and eastern regions of Peninsular Malaysia (Fig. 2b), sub-structuring further divided Cluster 1 (Fig. 2c) into two sub-clusters. Due to the strong admixture of alleles, Sub-cluster 1b (populations 20–27) harbours higher genetic diversity (Table 2, Fig. 2c). The observed pattern may be best explained by ocean current movements, in which the Straits of Malacca act as a channel connecting the Andaman Sea with South China Sea, flanked by Indonesian island of Sumatra and Peninsular Malaysia land mass, linking the mangrove ecosystems between the western and eastern regions through hydrological cycle^33,42^. The ocean current movements along the Straits of Malacca and South China Sea are highly dependent on the monsoon winds during the north-east (December–January) and south-west (June–July) monsoons^33^. This enables the mangroves propagules to float in ocean water for an extended period and follow the ocean currents, transported from the source to the sink^43^. Due to the shallow waters at water depth ~ 30 m, which is narrow in the southern part of the Straits of Malacca before meeting the South China Sea^42^, the propagules of either region travelled to the sink population and formed the observed admixed populations (Sub-cluster 1b).

Studies have shown that *R. apiculata* are strong dispersers with high propagules survivorship in seawater with capability of long-distance dispersal^44^. Nevertheless, the potential of long-distance dispersal can be limited due to several factors such as ocean circulations, wind, large distances, longevity and land barriers^14,44,45^. Peninsular Malaysia has been reported to serve as a land barrier to gene flow^15,45^, thus promoting genetic differentiation between *R. apiculata* populations, particularly those from the eastern and western regions of Peninsular Malaysia. Previous study suggested that limited gene dispersal likely played an important role in the evolutionary history of *Rhizophora* species, as frequent sea level fluctuations associated with climate changes would negatively impact their effective population sizes^9^. In addition, human activities such as logging and developments near mangrove habitat (as recorded in Table 2) might have created anthropogenic barriers that pose serious threats to gene flow. Such barriers can effectively split a species' range into isolated fragments, and dispersal from one population to another can prove difficult.

The low genetic diversity of *R. apiculata*, along with environmental fragility caused primarily by anthropogenic activities, can decrease its fitness and affect its long-term survival. Prior to this study, there was a lack of genetic and demographic information on *R. apiculata*, hampering local efforts to develop a conservation plan for the species. The newly generated genetic information will enable the formulation of comprehensive conservation guidelines for *R. apiculata* in Peninsular Malaysia. Since Sub-cluster 1b of the western cluster exhibited strong admixture pattern that harbours higher levels of genetic diversity, as a reservoir rich with admixed alleles from both western and eastern clusters, this sub-cluster deserves high priority for conservation. Besides, the selection of in situ conservation areas can be considered independently from the two main clusters with priority given to habitat protection to combat the potentially detrimental effects of inbreeding in the remaining *R. apiculata* populations.

## Methods

### Plant material collection and habitat condition survey

Sampling was carried out along sheltered coasts of Peninsular Malaysia, where *R. apiculata* grow abundantly in water zones where saltwater meets fresh water. We collected a total of 1,120 samples from 39 natural populations of *R. apiculata* between 2017 and 2018, with an average of 29 samples per population (Fig. 1, Table 2). Leaf samples were collected randomly from individual trees, cleaned, and kept in liquid nitrogen prior to DNA extraction. Genomic DNA of *R. apiculata* was extracted with a modified cetyl trimethylammonium bromide (CTAB) method^46^ and purified using High Pure PCR Template Preparation Kit ver. 20 (Roche, USA).

Besides, habitat condition survey was also carried out based on systematic observation of each population by boat and/or walking on foot, providing visual coverage on the anthropogenic activities of the surrounding area that could contribute to mangrove habitat destruction. We categorised the anthropogenic activities into two categories, ‘logged’ as logging for firewood, charcoal production and construction, and ‘developed’ as development for human settlements, aquaculture, ecotourism, road construction and land use for plantation, while undisturbed populations were recorded as ‘undisturbed’. Most of the *R. apiculata* sampling sites have experienced some form of anthropogenic activities either due to logging or development (Table 2). Several sites were considered undisturbed mostly due to poor accessibility and far from human settlements and activities.

### Microsatellite marker development and genotyping

The total RNA was extracted using Qiagen RNeasy kit (Qiagen, USA) and purified using the TURBO DNA-free kit (Ambion, USA). The quality of RNA sample was then checked using NanoDrop 2000 spectrophotometer (Thermo Scientific, USA). Transcriptome sequencing was carried out on an Illumina HiSeq 4000 sequencer (Illumina, USA) with default parameters at Novogene Bioinformatics Technology Co. Ltd. (Beijing, China). The raw data underwent quality checking, trimming, and assembling using FastQC^47^, Trimmomatic v0.32^48^ and Trinity v2.4.0^49^, respectively. Sequencing reads with many ambiguous (N) bases and more than 50% low-quality bases in raw reads were filtered out from the raw data set.

MicroSAtellite program (MISA, https://pgrc.ipk-gatersleben.de/misa)^50^ was used to identify microsatellite sequences of di-, tri, and tetranucleotide motifs. Markers were then designed with Primer3 (https://bioinfo.ut.ee/primer3-0.4.0/)^51^. We used the following criteria for marker selection: microsatellite motif length of ≤ 30 base pairs (bp); amplicon size of between 80 and 400 bp; and rejecting markers and amplicons with multiple mononucleotide repeat sequences. Based on these criteria, 60 primer pairs were synthesised and those that showed clear polymerase chain reaction (PCR) products on 1.5% agarose gel were fluorescently-labelled at the forward primer with HEX or 6-FAM. We performed PCR on a GeneAmp PCR System 9700 (Applied Biosystems, USA) in a final reaction volume of 10 μL. The PCR thermocycling parameters consisted of an initial denaturation step of 4 min at 94 °C followed by 40 cycles 94 °C for 1 min, 55 °C annealing temperature for 30 s, and 72 °C for 40 s, and the final extension step at 72 °C for 30 min. The PCR products were genotyped on an ABI 3130xl Genetic Analyzer (Applied Biosystems, USA) with ROX400 as the internal size standard. The alleles were scored using GeneMarker v2.6.4 (SoftGenetics, USA). The assembled contig sequences of the successfully developed microsatellite markers were assessed for reliability by blasting them to the *R. apiculata* genome sequence^22^ using BLASTN.

## Data analysis

Micro-Checker software was used to detect possible genotyping errors and null alleles^52^. We examined deviations from Hardy–Weinberg equilibrium (HWE) and linkage disequilibrium (LD) using Fisher’s exact test in Genetic Data Analysis (GDA) v1.1^53^. The sequential Bonferroni’s correction was conducted to adjust critical values for multiple comparisons, with a significance level of 5%^54^. Allelic frequencies for each locus in each population were obtained. The levels of genetic diversity within each population were estimated using Microsatellite Toolkit^55^ based on several parameters, including the average number of alleles per polymorphic locus (*A*_a_), effective number of alleles per polymorphic locus (*A*_e_), observed heterozygosity (*H*_o_), and expected heterozygosity (*H*_e_, Nei’s genetic diversity)^28^. Allelic richness (*R*_s_) was estimated using FSTAT v2.9.3^56^ and GDA v1.1, respectively. On the other hand, the inbreeding coefficient (*F*_IS_) was calculated using FSTAT v2.9.3^56^, and its significance was tested with GDA v1.1. Additional statistics, such as polymorphic information content (*PIC*) and Nei’s genetic distance (Nei’s *D*_A_)^57^, were calculated using POWERMARKER v3.25^58^.

We also analysed the levels of genetic differentiation in *R. apiculata* populations using the molecular fixation index (*F*_ST_)^59^, the divergence parameter (*R*_ST_)^38^, and Analysis of Molecular Variance (AMOVA), which were all determined by GenAlEx v6.5 at the significance level of 5%^60^. A Mantel test based on 9,999 permutations was carried out to examine if there was correlation between geographical distance and genetic differentiation (*F*_ST_, *R*_ST_ and *F*_ST_/1 − *F*_ST_), by analysing the Isolation-by-Distance (IBD) in GenAlEx v6.5. To infer the populations’ genetic structure, we applied a model-based Bayesian clustering method using STRUCTURE v2.3.1^61^. Dataset was explored using admixture model, which can detect structure among populations that are potentially similar due to shared ancestry or migration, with a burn-in of 250,000 steps followed by 850,000 Markov Chain Monte Carlo (MCMC) iterations. The StructureSelector software^62^ was then used to select and visualise the optimal number of clusters (*K*) in order to identify the highest level of genetic division hierarchy. Additionally, principal component analysis (PCA) was carried out using PCAGEN v1.2^63^ to assess the goodness-of-fit between simulated and real datasets, visualizing genetic distance and relatedness between populations in a two dimensional (2D) standard plot. To complement the analysis of the population structure, a UPGMA dendrogram was constructed based on Nei’s *D*_A_ using MEGA v5.0^64^. Bootstrap of 1,000 times was applied to acquire a reliable tree with correct branch topology.

## Supplementary information

Supplementary Information 1.

Supplementary Information 2.
