# Supplementary material for: Low genetic diversity indicating the threatened status of Rhizophora apiculata (Rhizophoraceae) in Malaysia: declined evolution meets habitat destruction
Source: Sci Rep. 2020 Nov 5;10:19112. doi: 10.1038/s41598-020-76092-4 (PMC7644706; doi:10.1038/s41598-020-76092-4)
Supplement: Supplementary file 1 — Supplementary Information 1. [file 41598_2020_76092_MOESM1_ESM.docx]

**Low genetic diversity indicating the threatened status of *Rhizophora apiculata* (Rhizophoraceae) in Malaysia: declined evolution meets habitat destruction**

Amelia Azman^1,2^, Kevin-Kit-Siong Ng^1^, Chin-Hong Ng^1^, Chai-Ting Lee^1^, Lee-Hong Tnah^1^, Nurul-Farhanah Zakaria^1^, Suhaila Mahruji^3,4^, Khairuddin Perdan^3^, Md-Zaidey Abdul-Kadir^3^, Acga Cheng^2,*^ and Soon-Leong Lee^1,*^

^1^ Genetics Laboratory, Forest Biotechnology Division, Forest Research Institute Malaysia, 52109 Kepong, Selangor Darul Ehsan, Malaysia

^2^ Functional Omics and Bioprocess Development Laboratory, Institute of Biological Sciences, Faculty of Science, University of Malaya, 50603 Kuala Lumpur, Malaysia

^3^ Forest Enforcement Division, Forestry Department of Peninsular Malaysia, Jalan Sultan

Salahuddin, 50660 Kuala Lumpur, Malaysia

^4^ Product Trade Section, Tariff Classification Division, Centre of Analysis for Industry and Custom Tariff, Chemistry Department of Malaysia, Jalan Sultan, 46661 Petaling Jaya, Selangor Darul Ehsan, Malaysia

*Corresponding author(s)

[leesl@frim.gov.my](mailto:leesl@frim.gov.my); [acgacheng@um.edu.my](mailto:acgacheng@um.edu.my)
